# Accurate indel prediction using paired-end short reads

**DOI:** 10.1186/1471-2164-14-132

**Published:** 2013-02-27

**Authors:** Dominik Grimm, Jörg Hagmann, Daniel Koenig, Detlef Weigel, Karsten Borgwardt

**Affiliations:** 1 Machine Learning and Computational Biology Research Group, Max Planck Institute for Developmental Biology and Max Planck Institute for Intelligent Systems, Tübingen, Germany; 2 Department of Molecular Biology, Max Planck Institute for Developmental Biology, Tübingen, Germany; 3 Center for Bioinformatics, Eberhard Karls Universität, Tübingen, Germany

**Keywords:** Next generation sequencing, Indel detection, Discriminative machine learning, Paired-end short reads, Split-read mapping

## Abstract

**Background:**

One of the major open challenges in next generation sequencing (NGS) is the accurate identification of structural variants such as insertions and deletions (indels). Current methods for indel calling assign scores to different types of evidence or counter-evidence for the presence of an indel, such as the number of split read alignments spanning the boundaries of a deletion candidate or reads that map within a putative deletion. Candidates with a score above a manually defined threshold are then predicted to be true indels. As a consequence, structural variants detected in this manner contain many false positives.

**Results:**

Here, we present a machine learning based method which is able to discover and distinguish true from false indel candidates in order to reduce the false positive rate. Our method identifies indel candidates using a discriminative classifier based on features of split read alignment profiles and trained on true and false indel candidates that were validated by Sanger sequencing. We demonstrate the usefulness of our method with paired-end Illumina reads from 80 genomes of the first phase of the 1001 Genomes Project ( http://www.1001genomes.org) in *Arabidopsis thaliana*.

**Conclusion:**

In this work we show that indel classification is a necessary step to reduce the number of false positive candidates. We demonstrate that missing classification may lead to spurious biological interpretations. The software is available at: http://agkb.is.tuebingen.mpg.de/Forschung/SV-M/.

## Background

The detection of genetic variation between individuals is a key challenge in current research in genome biology. This variation includes single nucleotide polymorphisms (SNPs), structural variants (SVs) and copy number variants (CNVs) such as deletions, insertions or duplications, as well as copy number invariant changes like translocations or inversions. SNPs are used extensively to link phenotypic traits with associated genotypes in genome-wide association studies (GWAS) [1] and to infer relationships in evolutionary studies [2,3]. SVs can provide additional insights into the genomic causes of phenotypic diversity [4,5]. Moreover, it is assumed that the total number of nucleotides spanned by SVs greatly exceeds that of SNPs in human and plants [6,7]. Hence SVs will be included more and more into these studies [6,8]. Furthermore, SVs are associated with different types of human diseases [5,9-13] and plant phenotypes [14,15]. Compared to SNP identification, the detection of larger divergent sequences remains a challenging task. We here present a machine learning approach to predict SVs based on NGS.

Traditionally, structural variants, in particular deletions and duplications, have been identified using array-based technologies (arrayCGH or SNP arrays) [16], but these strategies suffered from a limited size and localization resolution, which is dependent on the density of probes or known markers. With the advent of NGS methods, whole-genome studies became feasible. Small insertions and deletions (hereafter called indels) up to a few base pairs in length were called by sensitive alignment tools in the routine re-sequencing process [2,17,18]. However, the detection of larger structural variants based on depth-of-coverage (DOC) [19] or paired-end mapping (PEM) [20,21] methods could not reduce the SV size and localization resolution to the one base pair level. DOC [19] algorithms detect regions with absent (deletion) or significantly elevated (duplication) coverage, but are not able to determine the exact insertion location on the base pair level of the duplicated sequence. PEM [20,21] methods exploit the fact that the distance between the alignment locations of read pairs on a reference genome (the ‘insert size’ of the read pairs) usually follow a normal distribution. Clusters of read pairs mapping to the same genomic regions, whose distance is much shorter (longer) than expected can be explained by an insertion (deletion) in the newly sequenced individual compared to the reference genome. The standard deviation and the mean of the insert size distribution define the sensitivity of this method. Recently, so-called split-read mapping approaches (SRM) were introduced to pinpoint structural variants and especially indels in the genome correctly to the base pair level [22-24]. These methods use mapped-unmapped read pairs (MUR) from a paired-end alignment performed by existing short read mapping tools. The mapped partner serves as anchor to realign the unmapped partner using alignment algorithms allowing for long-range gaps in both the reference sequence (deletion) or the read (insertion). We will refer to the initial mapping which identifies MURs as first and the split read alignment as second mapping pass throughout this manuscript.

Deletions up to a few tens or hundreds of base pairs in length can be identified by array based, DOC and PEM approaches, while conventional short read alignments are designed to find only deletions of a few base pairs. In contrast, SRM predictions can in principle span the whole range of deletion lengths. However, the size of insertions is limited, and spurious alignments of the indel-flanking read parts might lead to multiple contradicting indel candidates. Both limitations directly depend on the read length and are thought to be counterbalanced by longer sequences from advanced technologies. Finally, deletions can be identified by limited *de novo* assembly methods, but they are not yet used routinely and require whole-genome alignments or close relative genomes for comparisons.

Though a large number of software packages for indel prediction from NGS data have been developed, application of these methods to identical data sets reveals little overlap [5]. This is caused by different indel identification strategies. To reduce the number of false positive indel candidates, SRM methods, such as Pindel [22], rely on conservative realignment strategies. Here, solely perfect and uniquely mapped reads are considered for further analysis. Moreover, the realignment of the unmapped partner has to be mismatch-free as well. These constraints reduce the set of possible indel candidates drastically. Existing SRM programs report an indel as soon as two independent reads support the same SV, and if their partner reads lie within concordant insert sizes [22,23]. Other methods use several alignment features as evidence of an indel. However, either relying on logical rules [22,24] or on generative probabilistic models [25], they require an empirically defined threshold, above which a candidate is a true SV. The most reliable way to verify indels is by capillary sequencing, but this is unfeasible for a genome-wide scan. Thus, to identify a comprehensive set of indels, a non-conservative mapping strategy is needed that takes non-unique and non-perfect mapping reads into account. Furthermore, to rate their trustworthiness, an evaluation method is needed which collects information about numerous alignment features from different approaches and automatically weighs their contributions.

Here we introduce an extended realignment split read strategy to identify a comprehensive set of indel candidates. A *de novo* machine learning method is applied to discriminate between ‘true’ and ‘false’ indel candidates based on more than 10 alignment features, which can be derived from any short read mapping tool. Its core is a support vector machine (SVM) [26], a discriminative classifier that is trained on diverse alignment information on indel examples validated by reliable Sanger sequencing. Our SVM approach avoids the step of defining thresholds for each feature by automatically learning them from Sanger validated training data. We show that a commonly used criterion, namely the number of split read pairs supporting the same indel, is not sufficient to distinguish true indels from false candidates, but that additional features can accurately predict bona fide SVs. Concomitantly, our method reports the contribution of each feature to this decision process. Our approach was applied to 80 genomes of *Arabidopsis thaliana*[2] and its validity demonstrated by recovering a highly similar population structure of the analyzed strains solely based on positively classified indels compared to taking SNP data as a basis.

## Results

### Indel candidate detection

We performed a custom split read alignment method to retrieve indel candidates from the *Arabidopsis thaliana* strain *ICE111* from phase I of the 1001 Genomes Project [2]. The read lengths ranged from 36 to 64 bp with an average sequencing depth of 21x. All mapped-unmapped read pairs (MURs) were retrieved from the available alignments from Cao *et al.*[2], which allowed 4 base pair differences between read and reference, of which at most 3 could be gaps. The mapped partner may have multiple alignment positions across the whole genome. Because of many ambiguous alignments due to the high repetitivity of centromeric sequences, all MURs within centromeres were excluded. The unmapped partners of the MURs were mapped against *Col-0* (TAIR8) in a 5,000 bp window downstream of the mapped partner using Gotoh’s alignment algorithm [27], allowing for long-range gaps as well as additional SNPs or few base pair-sized indels (Additional file 1). All best-scoring alignments were reported and indels with a minimum support of two reads constituted the indel candidate set to be further evaluated.

### Feature selection

The split read alignment approach identified 14,155 potential indel candidates for the *Arabidopsis thaliana* strain *ICE111*. We randomly selected 219 deletion and 43 insertion candidates across all chromosomes from this set and labeled them as true or false after Sanger sequencing. Thus, we retrieved two training sets. The training corpus for deletions consisted of 172 correctly and 47 falsely labeled examples and the training corpus for insertions of 33 true and 10 false ones. These sets were used to train a SVM [26].

**Figure 1:**
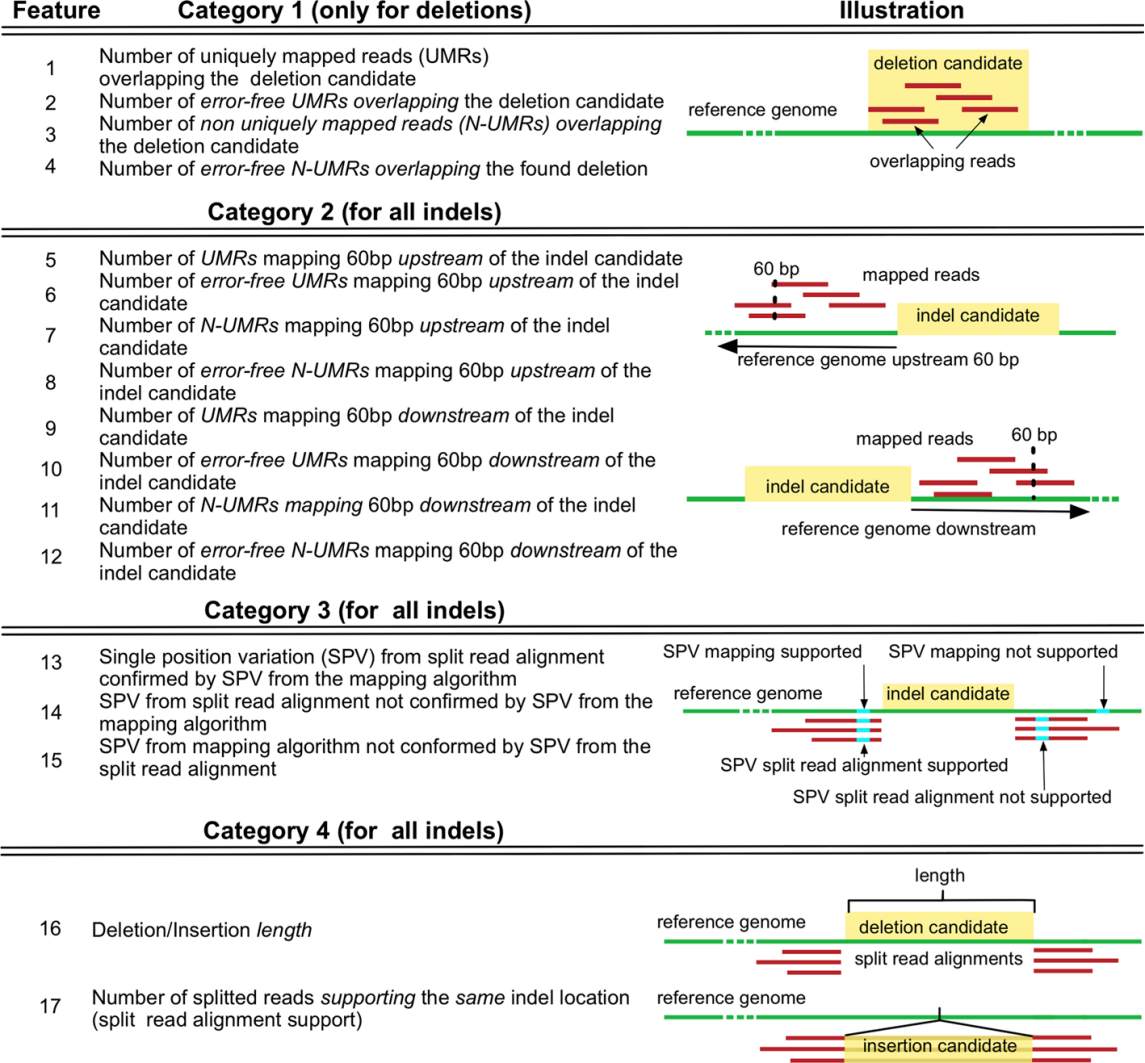
**Description and categorization of features.** The first category of features includes deletion candidates only, whereas categories 2-4 contain deletions and insertions.

Pindel [22] uses the number of split-read alignments supporting an indel with identical genomic coordinates as the only evidence for an indel. In a first study, only this alignment feature was used for classification (named *f1* training hereafter). The *f1*-based training was contrasted to the use of several alignment characteristics, 13 for insertions (*f13*) and 17 for deletions (*f17*). These features can be grouped into four main categories (Figure 1). The first considers the number of uniquely mapped reads (UMRs) and non-uniquely mapped reads (N-UMRs) overlapping the sequence space within a deletion. Since this is not determinable for insertion signatures, this feature is only available for deletions. The second group comprises the number of UMRs and N-UMRs 60 bp downstream as well as 60 bp upstream of the indel candidate to represent the coverages to the right and left where 60 bp reflects approximately the maximum read length. A ‘true’ deletion should show either zero or a low number of UMRs within the deleted region compared to the UMR-coverage up- and downstream thereof, whereas a certain number of N-UMRs might be tolerated. The third group of features examines the concordance of SNP and short indel calls detected by the two mapping passes (the short read mapping tool and our split read alignment step). Since these variations are compared to each other position-wise, short indels are considered as consecutive single position variants (SPVs). These features can be interpreted as a check if the aligned reads of the first mapping pass in the vicinity of an indel derive from the same haplotype as the split reads spanning the indel. The last category includes general attributes such as the indel length and the split read alignment support of identically-located indels.

### Discriminative training

**Figure 2:**
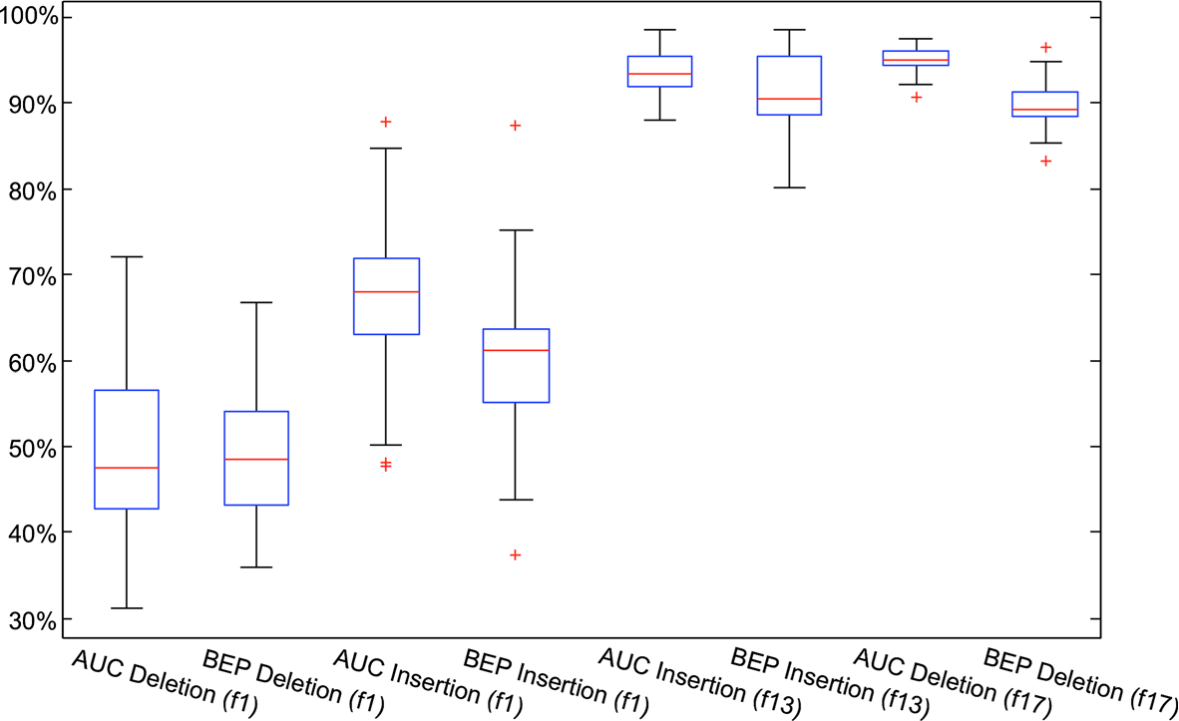
**Boxplots showing the performance for different feature sets.** The first 2 boxplots show the AUC and Spec-Sens-BEP of the single *f1* feature for deletions, the next two for insertions. The last four boxplots show the AUC and Spec-Sens-BEP using a set of 13 features for insertions (*f13*) and 17 features for deletions (*f17*).

We trained a SVM [26] using a simple-to-interpret linear kernel on all three sets of features (*f1*, *f13*, *f17*) performing a 10-fold cross-validation for deletions and a 5-fold cross-validation for insertions and repeated each cross-validation 100 times (Additional file 2). The resulting average area under the receiver operating characteristic curves (AUC) and average specificity-sensitivity-break-even-points (Spec-Sens-BEP) (Figure 2) suggest that the *f1*-based classification did not notably exceed the performance of a random guess for deletions (AUC = 49.3*%* ± 8.8*%*, Spec-Sens-BEP = 49.4*%*±7.5*%*) and performs slightly better for insertions (AUC = 67.0*%* ± 7.7*%*, Spec-Sens-BEP = 60.5*% *± 7.7*%*), whereas the use of 13 (AUC = 93.5*% *± 2.6*%*, Spec-Sens-BEP = 91.2*% *± 5.0*%*) and 17 features (AUC = 95.1*% *± 1.3*%*, Spec-Sens-BEP = 89.7*% *± 2.2*%*) reveal high concordance with the true classification.

**Figure 3:**
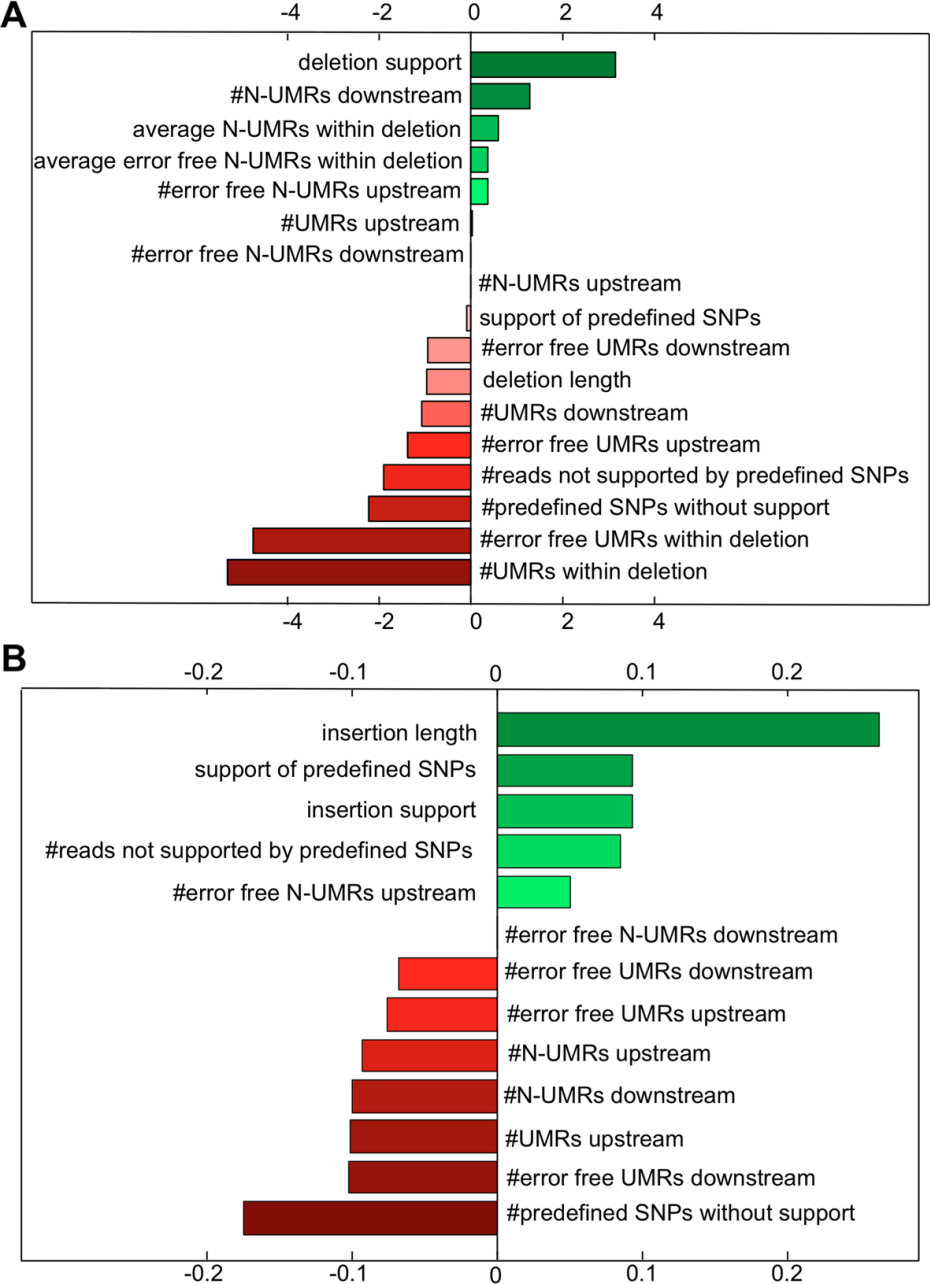
**Feature contributions.** Learned weights of a linear SVM of 17 features for deletions (**A**) and 13 features for insertions (**B**). A positive weight contributes to the support of an indel, whereas a negative weight contributes to the rejection.

The training of the SVM based on a linear kernel enabled us to identify the contributions of each feature to an indel prediction. Positive weights contribute to the support, and negative to the rejection of a candidate (Figure 3). Interestingly, the criteria for deletions and insertions notably differ from each other. While the strongest argument in favor for deletions is the number of SV supporting reads, it is the sequence length for insertions. Furthermore, the agreement of SPVs between the first and the second mapping pass contributes more to the acceptance of insertions, but is used as an indication against the trustworthiness of deletions. This effect might be explained by alignment errors by the first mapping pass close to deletions causing false positive SPV calls. Our classifier for insertions is trained on a dataset including 43 true insertions. Due to the limited size of this training dataset, it is to be expected that larger training datasets will further improve the prediction performance.

### Indel prediction

Applying our machine learning approach with the *f13*/*f17* feature set to indel candidates of strain *ICE111* positively classified 10,256 out of 13,547 deletions in total (76%), and 373 from 608 insertions (61%), respectively. The length of deletions ranged from 2 to 4,880 bp with a mean of 334 bp and a median of 12 bp. For insertions the length ranged from 2 to 5 bp with a mean and median of 4 bp. Thus, the SVM was capable to extract information from the defined features leading to falsifications of indel candidates. ’False’ SVs can be attributed to spurious mappings dependent on the length of the split read fragments or to multiple best-scoring alignments across the reference.

Next, we compared our predictions of indels in the strain *ICE111* to those identified by two versions of Pindel [22] (v0.1 and v0.24). The minimum length of deletions was set to 5 bp for all three sets, and the maximum deletion size constituted 5,000 bp due to the adjustable restriction of the alignment space. Pindel detected a total of 2,087 (v0.1) and 3,272 (v0.24) deletions larger than 5 bp. 99.8*%* (v0.1 and v0.24) of Pindel’s deletions were shared among all unclassified deletions of our approach. The SVM classification identified 220 (11%, v0.1) and 309 (10%, v0.24) false positive deletions among the Pindel candidates. Further, our Gotoh approach detected an additional set of 6,890 (v0.1) and 5,706 (v0.24) positively classified deletions. This can be explained by different mapping strategies. Pindel [22] in version 0.1 requires uniquely and error-free mapped reads and allows only split read alignments where the read partners are aligned within two times the average insertion size. On the contrary, our SVM approach follows a less conservative re-alignment strategy by analyzing non error-free and multiple best-scoring alignments of the same read. Moreover, we tolerated split read mappings anywhere within the alignment window, which results in a larger candidate set by allowing the detection of SVs in highly divergent regions. The subsequent classification compensates for these introduced levels of uncertainty. Indeed, Sanger sequencing revealed 14 (out of 219 validated) deletions, where the read partners showed discordant insert sizes. Furthermore, a 61bp deletion, which was included in the training set and falsified by Sanger sequencing, was reported by Pindel, but correctly classified as false by our SVM. This deletion would have proposed a potential frameshift in a coding region.

Due to general alignment restrictions, detecting insertions is limited in terms of their length. Aligning a short sequence against a long one by introducing a series of gaps into the long sequence at the same time leads necessarily to an inferior alignment score. Thus, Pindel (v0.1) and our approach share merely 15% of all insertions. Abyzov *et al.*[28] investigated exactly this problem and proposed an improved alignment algorithm called *AGE*. Applying this algorithm we could increase the number of shared insertions to 35%.

### Indel detection and prediction on 80 genomes

Next, we detected and classified indels in 80 accessions of *Arabidopsis thaliana* from the first phase of the 1001 Genomes Project [2]. The average coverage of strains was 17x. By using a 5,000 bp alignment window, on average ∼2,116 positive deletions and ∼69 positive insertions were reported per strain, the largest being 4,916 bp long. Combining similarly localized indels of identical length in different strains revealed 169,246 non-redundant deletions and 5,500 insertions in this population. Altogether, they span over 25 Mb in total. Almost half, ∼44%, were shared among more than one strain (Additional file 3).

We found 829 long-range deletions spanning at least one complete gene sequence of the TAIR8 annotation with an average allele frequency of ∼2.7 (ranging from 1 to 65). Of those deletions 101 were classified as whole gene losses if there was no unique read coverage within the deletion (at least 90% zero-covered positions) and sufficient coverage in the same-sized flanking regions (at least 90% non-zero-covered positions), as determined by the first mapping pass. Their average allele frequency was ∼4.4 (ranging up to 29). Spurious read mapping within the deletion, ambiguous split read alignments, gene translocations or heterozygous deletions could explain long-range deletions not meeting the aforementioned criteria.

As expected, only a minority (< 10*%*) of indels overlap with coding regions and have a potential deleterious effect on proteins (Additional file 4). Indels that do not alter the open reading frame of a gene outnumber those that do by almost two to one (Additional file 4). However, the prediction of amino acid or framshift changes has been performed for each SV separately without considering potential nearby SVs. It is known that additional nearby variants can compensate for frameshifts [29], thus the number of protein-changing SVs reported here might still be an overestimate.

### Population structure

**Figure 4:**
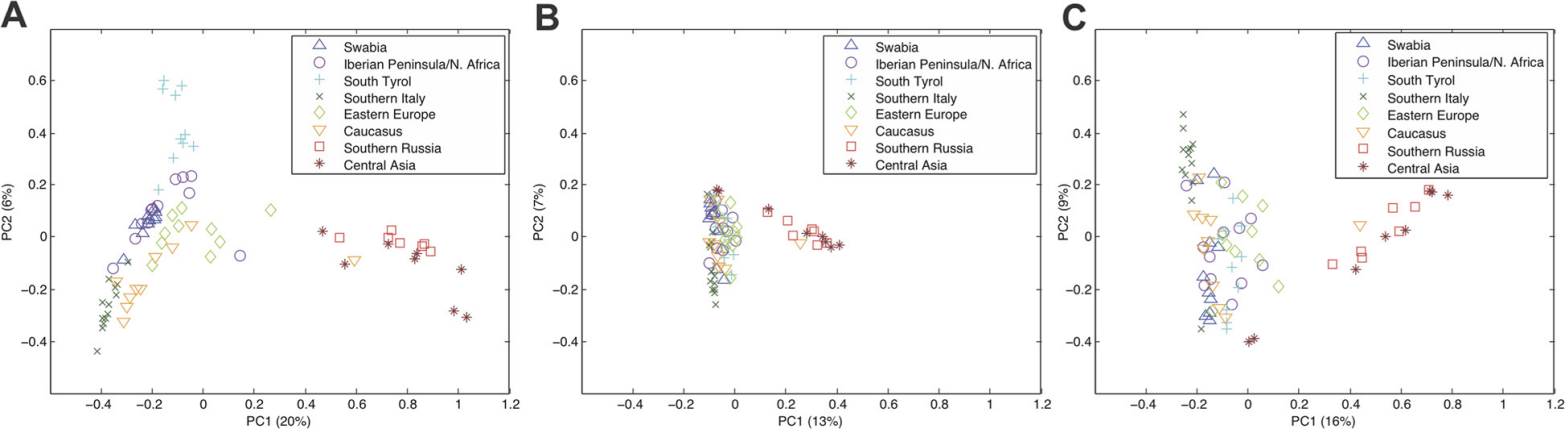
**Population structure of 80 accessions of the first phase of the 1001 genomes project.** (**A**) First two principle components (PC1 and PC2) of the covariance matrix of all positive predicted indels by our indel detection tool for 80 strains of *Arabidopsis thaliana*. (**B**) First two PCs of all detected indels by the tool Pindel (v0.1) and (**C**) by Pindel (v0.24).

To further assess our method, we attempted to recover the population structure of the 80 genomes with the predicted indels. To this end, three principal component analyses (PCA) [30] were performed: PCA1) on our 97,967 positively classified, non-private (shared by at least two different strains) indels (Figure 4A), PCA2) on the 37,294 non-private indels identified by the program Pindel [22] (v0.1) (Figure 4B), and PCA3) on the 53,417 non-private indels identified by the program Pindel (Figure 4C). PCA1 can successfully reconstruct the population structure, even slightly more distinctive as a PCA with non-private SNPs [2]. The first principal component distinguished the western and middle European accessions from the Caucasian and Russian individuals, explaining a variance of 20%. The second principle component with a variance of 6% was – as in Cao *et al.*[2] – not completely aligned with the latitude of the accessions. Interestingly, the outlier *Yeg-1* from the Caucasus found by Cao *et al.* was positioned near the South Russian and East Asian cluster in our analysis as well. PCA2 and PCA3 revealed that the reported indels of the program Pindel contain less information about population structure compared to our method. Furthermore, the clustering of the subpopulations in PCA1 is much more differentiable as in PCA2 and PCA3. The larger set of indels due to the more non-conservative re-alignment strategy and the removal of false indels (PCA1) seem to reflect the population structure more clearly, suggesting low rates of both false positives and false negatives.

## Discussion

We present a discriminative machine learning-based approach for detecting true structural variants among indel candidates. The key benefit of using a discriminative model is to learn to distinguish between true and false candidates based on a Sanger validated ground truth, thereby reducing the false positive rate among predicted indels.

We use our method on indel candidates generated via an exact Gotoh [27] re-alignment of paired-end reads, for which one partner could not be mapped. By considering multiply mapped reads on the whole genome and non-error free reads as well as accepting all mappings within the entire alignment window we receive a larger set of potential indel candidates. Consequently, this non-conservative proceeding increases the chances for finding more true positives, but on the other hand tends to identify more false positives as well. Due to that fact it is essential to accurately classify indel candidates using our machine learning approach.

Conceptually, our machine learning approach for true indel detection can be combined with any kind of alignment strategy and candidate generation scheme. Indeed, to be able to detect more insertions a different alignment method can be useful. With the Gotoh approach, shorter insertions are preferentially called than longer insertions in a pairwise alignment due to the reduced number of nucleotide matches (i.e. positive scores) the longer the insertion is. Abyzov *et al.* developed an alignment tool called AGE [28] to better call long insertions. Their method was used on the ICE111 genome in our framework and improved the overlap of insertions between Pindel and the Gotoh approach from 15% to 35%.

Current methods for indel scoring, which either rely on logical rules [22,24] or generative probabilistic models [25], have to manually define a threshold above which candidates are predicted to be true structural variants. Our machine learning approach avoids this step by automatically learning the threshold from the Sanger validated training data. Furthermore, all non-discriminative methods for scoring indel candidates have to solve the difficult task of how to weight different types of evidence for the occurrence of an indel. Unlike these methods, our discriminative approach automatically learns the weights of different features. In addition, the automatic weighting of features indicates which features are relevant and which ones are less relevant for indel detection. From our results, we can confirm that our discriminative indel detection benefits from combining several features [25].

The features we selected contain information that can reliably distinguish between true indels and false candidates as demonstrated by the consistent reconstruction of population structure based on true predicted indels. Furthermore, we showed that tools not relying upon a classification step may lead to spurious biological interpretations. Here, Pindel [22] identified a deletion candidate causing a potential frameshift, which our post-classification method predicted to be a false one; this prediction was confirmed by one of the Sanger sequences. The classifier was trained on a corpus of reads from a paired-end library sequenced to 21-fold coverage. Our method is robust to changes in fold coverage if the features that are derived from the read alignments, scale linearly with or are independent of sequencing depth. It is expected, that longer reads will improve our strategy since the longer the indel-flanking read sequences the less ambiguous split read alignments will be retrieved. The feature normalization we perform accounts for this fold change. To apply our method in different species, one would need to create a new Sanger validated dataset to account for its particular genomic properties such as the degree of heterozygosity or repetitiveness. However, to circumvent laborious Sanger sequencing, the increasing number of *de novo* assembled genomes or structural variant databases could serve as an alternative and extensive ground truth in future studies.

The software, Sanger validated training data and all annotated indels for the 80 genomes are available at http://agkb.is.tuebingen.mpg.de/Forschung/SV-M/.

## Conclusion

We showed that accurate indel detection consists of two steps – the realignment of unmapped reads and the post-classification of detected candidates. Methods that rely predominantly on re-alignment strategies often contain a large number of false detected indels. We used a nonconservative re-alignment strategy (e.g. allowing multiply mapped reads) to enrich the number of candidates and applied a discriminative machine learning-based approach to then classify indel candidates into true and false ones. We achieved a classification accuracy of 95.1*% *± 1.3*%* for deletions and 93.5*% *± 2.6*%* for insertions. Furthermore, we showed that indel classification reduces the number of false candidates significantly and that missing classification may lead to spurious biological interpretations such as false frame shifts or gene losses.

## Methods

### Support Vector Machine

A Support Vector Machine (SVM) [26] is a classifier which uses a hyperplane for classification. A SVM deals with a binary classification problem. We assume that we are given a set of data points $D=\{(x_{1},y_{1}),\ldots ,(x_{m},y_{m})\}$, where $x_{i}\in R^{d}$. The label *y*_*i*_ of a point *x*_*i*_ describes whether the point is in the negative (*y*_*i *_= −1) or the positive class (*y*_*i *_= 1). A SVM tries to separate the set D into a positive and a negative class by using a hyperplane. The process of finding the hyperplane is referred to as training. The hyperplane then defines a decision function to find the unknown label *y*_*i*_ for a new data point *x*_*i*_. We use a soft-margin variant of the SVM, the C-SVM which is maximizing the margin and is minimizing the training error at the same time. The C-SVM uses a penalty factor *C*, which penalizes wrongly classified points in the training set. Our developed software is written in C/C++ and uses libsvm [31], a library for Support Vector Machines.

### Classifier training

To train a classifier using a C-SVM and a linear kernel we labeled a set of detected indel candidates by reliable Sanger sequencing as true (positive class) and false indels (negative class). We split the validated corpus into a training and testing set. The training set was used to learn the discriminative model whereas the testing set measured the accuracy of the model. We counted a correctly classified test point as a true positive (*TP*) if the test point corresponded to the positive class and as true negative (*TN*) if the test point corresponded to the negative class. A false negative (*FN*) is a positive test point that is classified into the negative class. Correspondingly, a false positive (*FP*) is a negative test point that is classified into the positive class. For the actual training we selected 13 features for insertions and 17 for deletions derived from the split read alignment profiles (Figure 1). To train the SVM with these features, we normalized the data within the interval [0,1] and used a 10-fold cross-validation for deletions and a 5-fold cross-validation for insertions (Additional file 2). We repeated each cross-validation experiment 100 times. After cross-validation the best performing soft-margin parameter *C* value is 10 for deletions and 0.10 for insertions. To measure the performance we computed the area under (AUC) the receiver operation characteristic (ROC) curve and the specificity-sensitivity-break-even point (Spec-Sens-BEP). The ROC curve is the fraction of the *TP* over all positives *TP* + *FN*(sensitivity or true positive rate (*TPR*)) against the fraction of *TN* over all negatives *TN* + *FP*(specificity or true negative rate (*TNR*)). The Spec-Sens-BEP is the point where the *TPR* is equal to the *TNR*.

### Split read alignment

To detect indels we used the short read alignments of 80 strains from *Arabidopsis thaliana* against its reference genome *Col-0* provided by Cao *et al.*[2]. We parsed all read pairs, from which only one partner could be mapped (MURs). The mapped partner of a MUR may contain mismatches and may have multiple mapping positions across the whole genome. The mapped partner served as an anchor point. We considered a sequence window of length 5000 bp downstream of the anchor. Depending on whether the mapped read is located at the forward or backward strand we have to span the alignment window upstream or downstream. Using an exact Gotoh [27] alignment we aligned the unmapped read within this window against the reference (Additional file 1). The Gotoh [27] alignment is based on the Smith-Waterman [32] algorithm to compute a local pair-wise alignment between two sequences *a* and *b* using affine gap costs. Gotoh described in his work how to use dynamic programming to compute an alignment with affine gap costs in O(mn), where *m* and *n* are the lengths of sequences *a* and *b*. As alignment matrix we used the *NUC4.2* scoring matrix ( ftp://ftp.ncbi.nih.gov/blast/matrices/), which scores a match with 5 and a mismatch with -4. A gap opening is scored with -10, whereas a gap extension scores with zero. Alignments with a score less than the maximal alignment score minus 30 were discarded allowing for up to 7 mismatches or 3 gap openings. If the alignment contains a sequence of at least two consecutive gaps either in the unmapped read or in the reference, a possible indel location was reported. For short reads this single split read alignment is not a strong indicator of an indel due to similar regions and alignment errors. Hence we considered a location as a possible indel candidate only if we found a second independent split read alignment that supported the same location. Furthermore, each fragment of the split read alignment (left and right part of the read compared to the indel) had to be at least 8 bp long. We then used a pre-trained SVM [26] to predict whether an indel candidate is a true indel.

### Population structure

Novembre *et al.*[30] showed that the eigenvectors of the SNP covariance matrix reflect the population structure. We here used an indel covariance matrix. For this purpose, we combined identical or few base pair-shifted indels of the same length among different strains into an *MxN* matrix, where *M* is the number of strains and *N* the number of indels. All deletions and insertions with an SV frequency of at least two among all strains were encoded with 1 and -1, respectively. The absence of an indel was specified with zero. To compute the underlying population structure for all eighty genomes for the first phase of the 1001 genomes project for *Arabidopsis thaliana*[2] we conducted a principle component analysis (PCA) using a custom Matlab script.

## Abbreviations

NGS: Next Generation Sequencing; SVM: Support Vector Machine; MUR: mapped-unmapped read pair; SV: structural variant; SNP: single nucleotide polymorphism; GWA: genome wide association; DOC: depth-of-coverage; PEM: paired-end mapping; SRM: split-read mapping; UMR: uniquely mapped read; N-UMR: non-uniquely mapped read; SPV: single position variant; AUC: area under the curve; Spec-Sens-BEP: specificity-sensitivity-break-even-point; PCA: principle component analyses; PC: principle component; ROC: receiver operation characteristic; TN: true negative; TP: true positive; FN: false negative; FP: false positive; TPR: true positive rate; TNR: true negative rate.

## Competing interests

The authors declare that they have no competing financial interests.

## Authors’ contributions

DG, JH, DW and KB conceived the study; DG implemented the methods; DG and JH analyzed the data; DK performed the Sanger sequencing; DG, JH and KB wrote the paper with contributions from all authors. All authors read and approved the final manuscript.

## Supplementary Material

Additional file 1 **Split read re-alignment approach.** The mapped read serves as anchor for the re-alignment of the unmapped read. Using an exact Gotoh alignment the unmapped read is aligned against the reference. If the read can be split in at least 2 fragments it is an indication of a possible deletion location (A). If the reference can be split in at least 2 fragments it is an indication of a possible insertion location (B). Click here for file

Additional file 2 **Illustration of the k-fold cross-validation process.** The positively and negatively labeled examples are split into *k* distinct training and test sets *t*_*i*_ and *e*_*i*_, where 1 ≤* i *≤* k*. To determine the best performing *C* value each training set *t*_*i*_ is split into sub-training and sub-testing sets *t*_*s *_and *e*_*s*_, where 1 ≤* s *≤* k*. On basis of these subsets the SVM is trained several times using *C* values ranging from 10^−5^ to 10^5^. The *C* value with the highest Spec-Sens-BEP is used to train the SVM with the entire training set *t*_*i*_. The test set *e*_*i*_ is used to test the performance of the trained SVM by computing the Spec-Sens-BEP. These steps are repeated *k* times. Finally the average Spec-Sens-BEP is computed. Click here for file

Additional file 3 **Allele frequency of deletions and insertions in 80 genomes.** The allele frequencies for deletions (A) and insertions (B), for which there was sufficient read information (see Cao *et al.*[2] for criteria) in all 80 strains at or 10bp surrounding the indel. They are split by functional annotation classes (obtained from TAIR8). The bars indicate the fractions of indels of each annotation class per allele frequency from all indels of the corresponding annotation class (the total number of indels in an annotation class is denoted in parentheses in the legend labels). Indels overlapping with features of different annotation classes were classified based on following priorities: CDS > UTR > intron > transposon > intergenic. Indels overlapping with coding features were classified based on following priorities: gene loss (for deletions only) > start codon change or loss > splice site change or loss > premature stop codon > stop codon change or loss > in-frame. In-frame indels do not change the frame of the coding sequence. Annotations were performed on each indel without taking into account putative compensating indels or SNPs nearby. Click here for file

Additional file 4 **TAIR8 annotation classes.** Annotation classes of 169,246 deletions (A) and 5,500 insertions (B) in 80 genomes of *Arabidopsis thaliana*. For explanation of the classification scheme, see legend of Additional file 3. (C) Fractions of indels overlapping with coding sequences and overlapping with nongenic regions from all indels in corresponding classes, split by the remainder of the division of their lengths by 3. In genic regions, it is the frame of the CDS downstream of the indel. Structural variations with a length dividable by 3 in coding regions do not alter the open reading frame and are more likely to be synonymous. Click here for file

## References

[B1] Atwell S Huang YS Vilhjálmsson BJ Willems G Horton M Li Y Meng D Platt A Tarone AM Hu TT Jiang R Muliyati NW Zhang X Amer MA Baxter I Brachi B Chory J Dean C Debieu M de Meaux J Ecker JR Faure N Kniskern JM Jones JDG Michael T Nemri A Roux F Salt DE Tang C Todesco M Traw MB Weigel D Marjoram P Borevitz JO Bergelson J Nordborg M Genome-wide association study of 107 phenotypes in Arabidopsis thaliana inbred lines Nature 2010 465 7298 627 631 [ http://www.ncbi.nlm.nih.gov/pubmed/20336072]. [PMID:20336072] 10.1038/nature08800 20336072 PMC3023908

[B2] Cao J Schneeberger K Ossowski S Günther T Bender S Fitz J Koenig D Lanz C Stegle O Lippert C Wang X Ott F Müller J Alonso-Blanco C Borgwardt K Schmid KJ Weigel D Whole-genome sequencing of multiple Arabidopsis thaliana populations Nat Genet 2011 43 10 956 963 [ http://www.ncbi.nlm.nih.gov/pubmed/21874002]. [PMID:21874002] 10.1038/ng.911 21874002

[B3] Platt A Horton M Huang YS Li Y Anastasio AE Mulyati NW Ågren J Bossdorf O Byers D Donohue K Dunning M Holub EB Hudson A Le Corre V Loudet O Roux F Warthmann N Weigel D Rivero L Scholl R Nordborg M Bergelson J Borevitz JO The scale of population structure in Arabidopsis thaliana PLoS Genet 2010 6 2 e1000843 [ http://dx.doi.org/10.1371/journal.pgen.1000843] 10.1371/journal.pgen.1000843 20169178 PMC2820523

[B4] Korbel JO Urban AE Affourtit JP Godwin B Grubert F Simons JF Kim PM Palejev D Carriero NJ Du L Taillon BE Chen Z Tanzer A Saunders ACE Chi J Yang F Carter NP Hurles ME Weissman SM Harkins TT Gerstein MB Egholm M Snyder M Paired-end mapping reveals extensive structural variation in the human genome Science (New York, N.Y.) 2007 318 5849 420 426 [ http://www.ncbi.nlm.nih.gov/pubmed/17901297]. [PMID:17901297] 10.1126/science.1149504 PMC2674581 17901297

[B5] Mills RE Walter K Stewart C Handsaker RE Chen K Alkan C Abyzov A Yoon SC Ye K Cheetham RK Chinwalla A Conrad DF Fu Y Grubert F Hajirasouliha I Hormozdiari F Iakoucheva LM Iqbal Z Kang S Kidd JM Konkel MK Korn J Khurana E Kural D Lam HYK Leng J Li R Li Y Lin C Luo R Mu XJ Nemesh J Peckham HE Rausch T Scally A Shi X Stromberg MP Stutz AM Urban AE Walker JA Wu J Zhang Y Zhang ZD Batzer MA Ding L Marth GT McVean G Sebat J Snyder M Wang J Ye K Eichler EE Gerstein MB Hurles ME Lee C McCarroll SA Korbel JO Mapping copy number variation by population-scale genome sequencing Nature 2011 470 7332 59 65 [ http://dx.doi.org/10.1038/nature09708] 10.1038/nature09708 21293372 PMC3077050

[B6] Alkan C Coe BP Eichler EE Genome structural variation discovery and genotyping Nat Rev Genet 2011 12 5 363 376 [ http://dx.doi.org/10.1038/nrg2958] 10.1038/nrg2958 21358748 PMC4108431

[B7] Gan X Stegle O Behr J Steffen JG Drewe P Hildebrand KL Lyngsoe R Schultheiss SJ Osborne EJ Sreedharan VT Kahles A Bohnert R Jean G Derwent P Kersey P Belfield EJ Harberd NP Kemen E Toomajian C Kover PX Clark RM Ratsch G Mott R Multiple reference genomes and transcriptomes for Arabidopsis thaliana Nature 2011 477 7365 419 423 [ http://dx.doi.org/10.1038/nature10414] 10.1038/nature10414 21874022 PMC4856438

[B8] Medvedev P Stanciu M Brudno M Computational methods for discovering structural variation with next-generation sequencing Nat Methods 2009 6 11 Suppl S13—20 [ http://www.ncbi.nlm.nih.gov/pubmed/19844226]. [PMID:19844226] 19844226 10.1038/nmeth.1374

[B9] Sebat J Lakshmi B Malhotra D Troge J Lese-Martin C Walsh T Yamrom B Yoon S Krasnitz A Kendall J Leotta A Pai D Zhang R Lee Y Hicks J Spence SJ Lee AT Puura K Lehtimäki T Ledbetter D Gregersen PK Bregman J Sutcliffe JS Jobanputra V Chung W Warburton D King M Skuse D Geschwind DH Gilliam TC Ye K Wigler M Strong association of *de novo* copy number mutations with autism Science (New York, N.Y.) 2007 316 5823 445 449 [ http://www.ncbi.nlm.nih.gov/pubmed/17363630]. [PMID: 17363630] 10.1126/science.1138659 PMC2993504 17363630

[B10] McCarroll SA Altshuler DM Copy-number variation and association studies of human disease Nat Genet 2007 39 7 Suppl S37—42 [ http://www.ncbi.nlm.nih.gov/pubmed/17597780]. [PMID:17597780] 17597780 10.1038/ng2080

[B11] McCarroll SA Huett A Kuballa P Chilewski SD Landry A Goyette P Zody MC Hall JL Brant SR Cho JH Duerr RH Silverberg MS Taylor KD Rioux JD Altshuler D Daly MJ Xavier RJ Deletion polymorphism upstream of IRGM associated with altered IRGM expression and Crohn’s disease Nat Genet 2008 40 9 1107 1112 [ http://www.ncbi.nlm.nih.gov/pubmed/19165925]. [PMID:19165925] 10.1038/ng.215 19165925 PMC2731799

[B12] McCarthy MI Abecasis GR Cardon LR Goldstein DB Little J Ioannidis JPA Hirschhorn JN Genome-wide association studies for complex traits: consensus, uncertainty and challenges Nat Rev Genet 2008 9 5 356 369 [ http://dx.doi.org/10.1038/nrg2344] 10.1038/nrg2344 18398418

[B13] Stefansson H Rujescu D Cichon S Pietiläinen OPH Ingason A Steinberg S Fossdal R Sigurdsson E Sigmundsson T Buizer-Voskamp JE Hansen T Jakobsen KD Muglia P Francks C Matthews PM Gylfason A Halldorsson BV Gudbjartsson D Thorgeirsson TE Sigurdsson A Jonasdottir A Jonasdottir A Bjornsson A Mattiasdottir S Blondal T Haraldsson M Magnusdottir BB Giegling I Möller H Hartmann A Shianna KV Ge D Need AC Crombie C Fraser G Walker N Lonnqvist J Suvisaari J Tuulio-Henriksson A Paunio T Toulopoulou T Bramon E Di Forti M Murray R Ruggeri M Vassos E Tosato S Walshe M Li T Vasilescu C Mühleisen TW Wang AG Ullum H Djurovic S Melle I Olesen J Kiemeney LA Franke B Sabatti C Freimer NB Gulcher JR Thorsteinsdottir U Kong A Andreassen OA Ophoff RA Georgi A Rietschel M Werge T Petursson H Goldstein DB Nöthen MM Peltonen L Collier DA St Clair D Stefansson K Large recurrent microdeletions associated with schizophrenia Nature 2008 455 7210 232 236 [ http://www.ncbi.nlm.nih.gov/pubmed/18668039]. [PMID:18668039] 10.1038/nature07229 18668039 PMC2687075

[B14] Johanson U West J Lister C Michaels S Amasino R Dean C Molecular analysis of FRIGIDA, a major determinant of natural variation in Arabidopsis flowering time Science 2000 290 5490 344 347 [ http://www.sciencemag.org/content/290/5490/344.abstract] 10.1126/science.290.5490.344 11030654

[B15] Michaels SD He Y Scortecci KC Amasino RM Attenuation of FLOWERING LOCUS C activity as a mechanism for the evolution of summer-annual flowering behavior in Arabidopsis Proc Nat Acad Sci 2003 100 17 10102 10107 [ http://www.pnas.org/content/100/17/10102.abstract] 10.1073/pnas.1531467100 12904584 PMC187779

[B16] Carter NP Methods and strategies for analyzing copy number variation using DNA microarrays Nat Genet 2007 39 S16 S21 10.1038/ng2028 17597776 PMC2697494

[B17] Ossowski S Schneeberger K Clark RM Lanz C Warthmann N Weigel D Sequencing of natural strains of Arabidopsis thaliana with short reads Genome Res 2008 18 12 2024 2033 [ http://www.ncbi.nlm.nih.gov/pubmed/18818371]. [PMID:18818371] 10.1101/gr.080200.108 18818371 PMC2593571

[B18] Schneeberger K Hagmann J Ossowski S Warthmann N Gesing S Kohlbacher O Weigel D Simultaneous alignment of short reads against multiple genomes Genome Biol 2009 10 R98 [ http://genomebiology.com/content/10/9/R98] 10.1186/gb-2009-10-9-r98 19761611 PMC2768987

[B19] Campbell PJ Stephens PJ Pleasance ED O’Meara S Li H Santarius T Stebbings LA Leroy C Edkins S Hardy C Teague JW Menzies A Goodhead I Turner DJ Clee CM Quail MA Cox A Brown C Durbin R Hurles ME Edwards PAW Bignell GR Stratton MR Futreal PA Identification of somatically acquired rearrangements in cancer using genome-wide massively parallel paired-end sequencing Nat Genet 2008 40 6 722 729 [ http://dx.doi.org/10.1038/ng.128] 10.1038/ng.128 18438408 PMC2705838

[B20] Tuzun E Sharp AJ Bailey JA Kaul R Morrison VA Pertz LM Haugen E Hayden H Albertson D Pinkel D Olson MV Eichler EE Fine-scale structural variation of the human genome Nat Genet 2005 37 7 727 732 [ http://dx.doi.org/10.1038/ng1562] 10.1038/ng1562 15895083

[B21] Lee S Cheran E Brudno M A robust framework for detecting structural variations in a genome Bioinformatics 2008 24 13 i59 i67 [ http://bioinformatics.oxfordjournals.org/content/24/13/i59.abstract] 10.1093/bioinformatics/btn176 18586745 PMC2718654

[B22] Ye K Schulz MH Long Q Apweiler R Ning Z Pindel: a pattern growth approach to detect break points of large deletions and medium sized insertions from paired-end short reads Bioinformatics (Oxford, England) 2009 25 21 2865 2871 [ http://www.ncbi.nlm.nih.gov/pubmed/19561018]. [PMID:19561018] 10.1093/bioinformatics/btp394 PMC2781750 19561018

[B23] Zhang J Wu Y SVseq: an approach for detecting exact breakpoints of deletions with low-coverage sequence data Bioinformatics 2011 27 23 3228 3234 [ http://bioinformatics.oxfordjournals.org/content/27/23/3228.abstract] 10.1093/bioinformatics/btr563 21994222

[B24] Zhang ZD Du J Lam H Abyzov A Urban AE Snyder M Gerstein M Identification of genomic indels and structural variations using split reads. BMC, journal=Genomics 2011 12 375 [ http://www.ncbi.nlm.nih.gov/pubmed/21787423]. [PMID:21787423] 10.1186/1471-2164-12-375 PMC3161018 21787423

[B25] DePristo MA Banks E Poplin R Garimella KV Maguire JR Hartl C Philippakis AA del Angel G Rivas MA Hanna M McKenna A Fennell TJ Kernytsky AM Sivachenko AY Cibulskis K Gabriel SB Altshuler D Daly MJ A framework for variation discovery and genotyping using next-generation DNA sequencing data Nat Genet 2011 43 5 491 498 [ http://dx.doi.org/10.1038/ng.806] 10.1038/ng.806 21478889 PMC3083463

[B26] Noble WS What is a support vector machine? Nat Biotech 2006 24 12 1565 1567 [ http://dx.doi.org/10.1038/nbt1206-1565] 10.1038/nbt1206-1565 17160063

[B27] Gotoh O An improved algorithm for matching biological sequences J Mol Biol 1982 162 3 705 708 [ http://www.ncbi.nlm.nih.gov/pubmed/7166760]. [PMID:7166760] 10.1016/0022-2836(82)90398-9 7166760

[B28] Abyzov A Gerstein M AGE: defining breakpoints of genomic structural variants at single-nucleotide resolution, through optimal alignments with gap excision Bioinformatics 2011 27 5 595 603 [ http://bioinformatics.oxfordjournals.org/content/27/5/595.abstract] 10.1093/bioinformatics/btq713 21233167 PMC3042181

[B29] Schneeberger K Ossowski S Ott F Klein JD Wang X Lanz C Smith LM Cao J Fitz J Warthmann N Henz SR Huson DH Weigel D Reference-guided assembly of four diverse Arabidopsis thaliana genomes Proc Nat Acad Sci 2011 108 25 10249 10254 [ http://www.pnas.org/content/108/25/10249.abstract] 10.1073/pnas.1107739108 21646520 PMC3121819

[B30] Novembre J Johnson T Bryc K Kutalik Z Boyko AR Auton A Indap A King KS Bergmann S Nelson MR Stephens M Bustamante CD Genes mirror geography within Europe Nature 2008 456 7218 98 101 [ http://www.ncbi.nlm.nih.gov/pubmed/18758442]. [PMID:18758442] 10.1038/nature07331 18758442 PMC2735096

[B31] Chang C Lin C LIBSVM: A library for support vector machines ACM Trans Intell Syst Technol 2011 2 3 27:1 27:27 [ http://doi.acm.org/10.1145/1961189.1961199]

[B32] Smith TF Waterman MS Identification of common molecular subsequences J Mol Biol 1981 147 195 197 [ http://www.ncbi.nlm.nih.gov/pubmed/7265238]. [PMID:7265238] 10.1016/0022-2836(81)90087-5 7265238

