# Supplementary figures and images for: Accurate indel prediction using paired-end short reads
Source: BMC Genomics. 2013 Feb 27;14:132. doi: 10.1186/1471-2164-14-132 (PMC3614465; doi:10.1186/1471-2164-14-132)

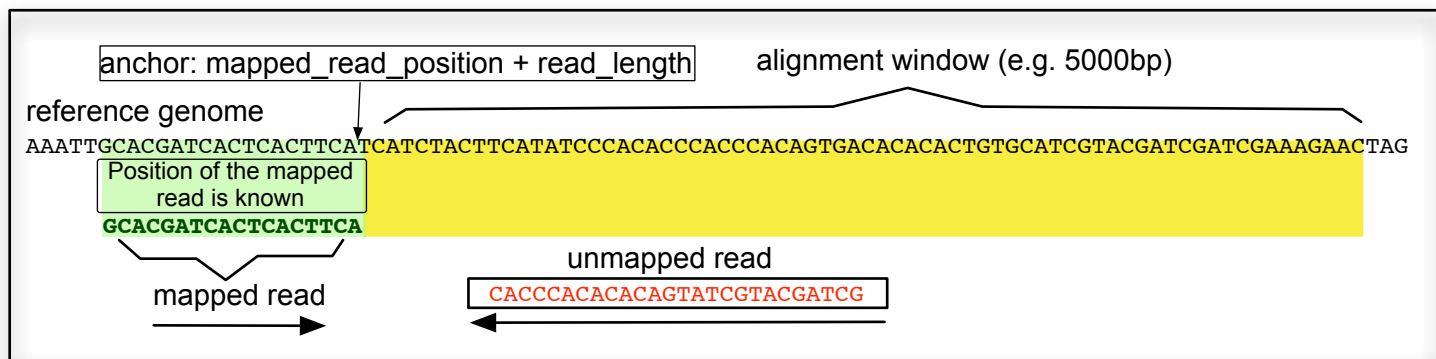

Gotoh alignment

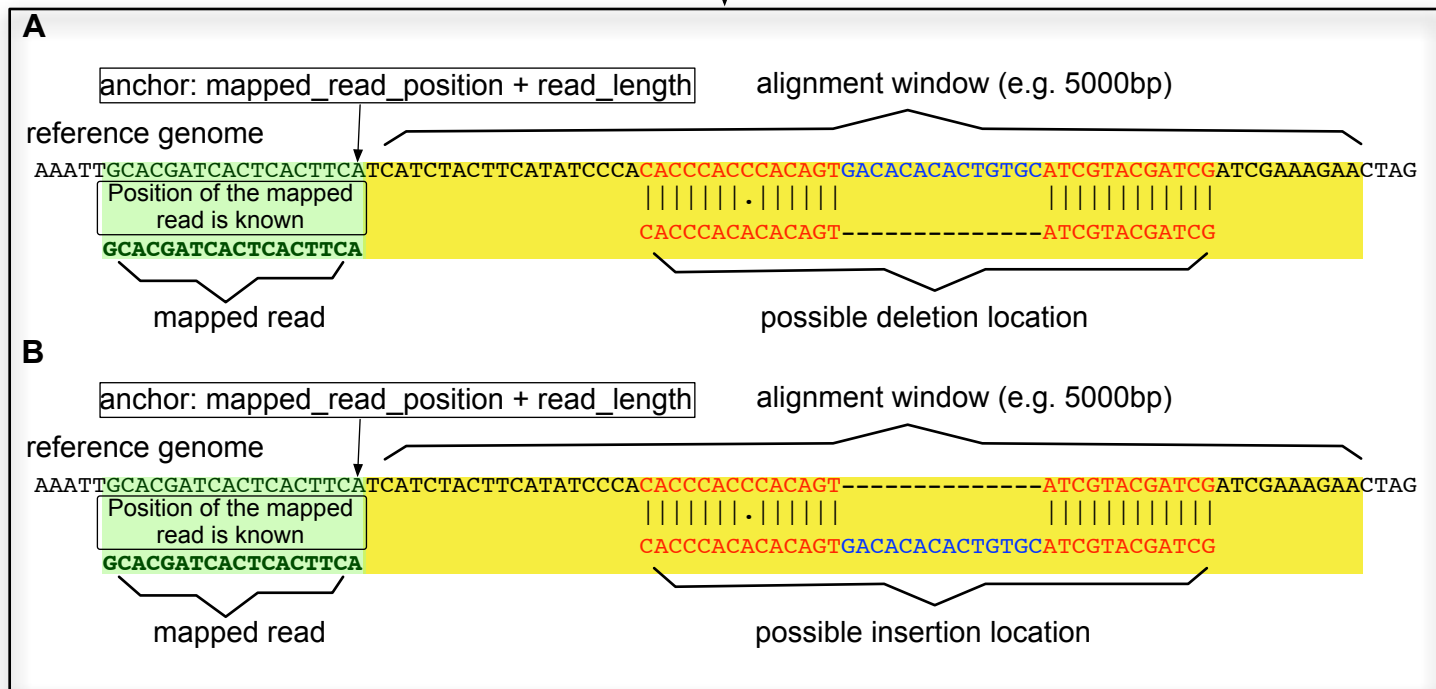

Supplement: Additional file 1 — Split read re-alignment approach. The mapped read serves as anchor for the re-alignment of the unmapped read. Using an exact Gotoh alignment the unmapped read is aligned against the reference. If the read can be split in at least 2 fragments it is an indication of a possible deletion location (A). If the reference can be split in at least 2 fragments it is an indication of a possible insertion location (B). [file 1471-2164-14-132-S1.pdf]

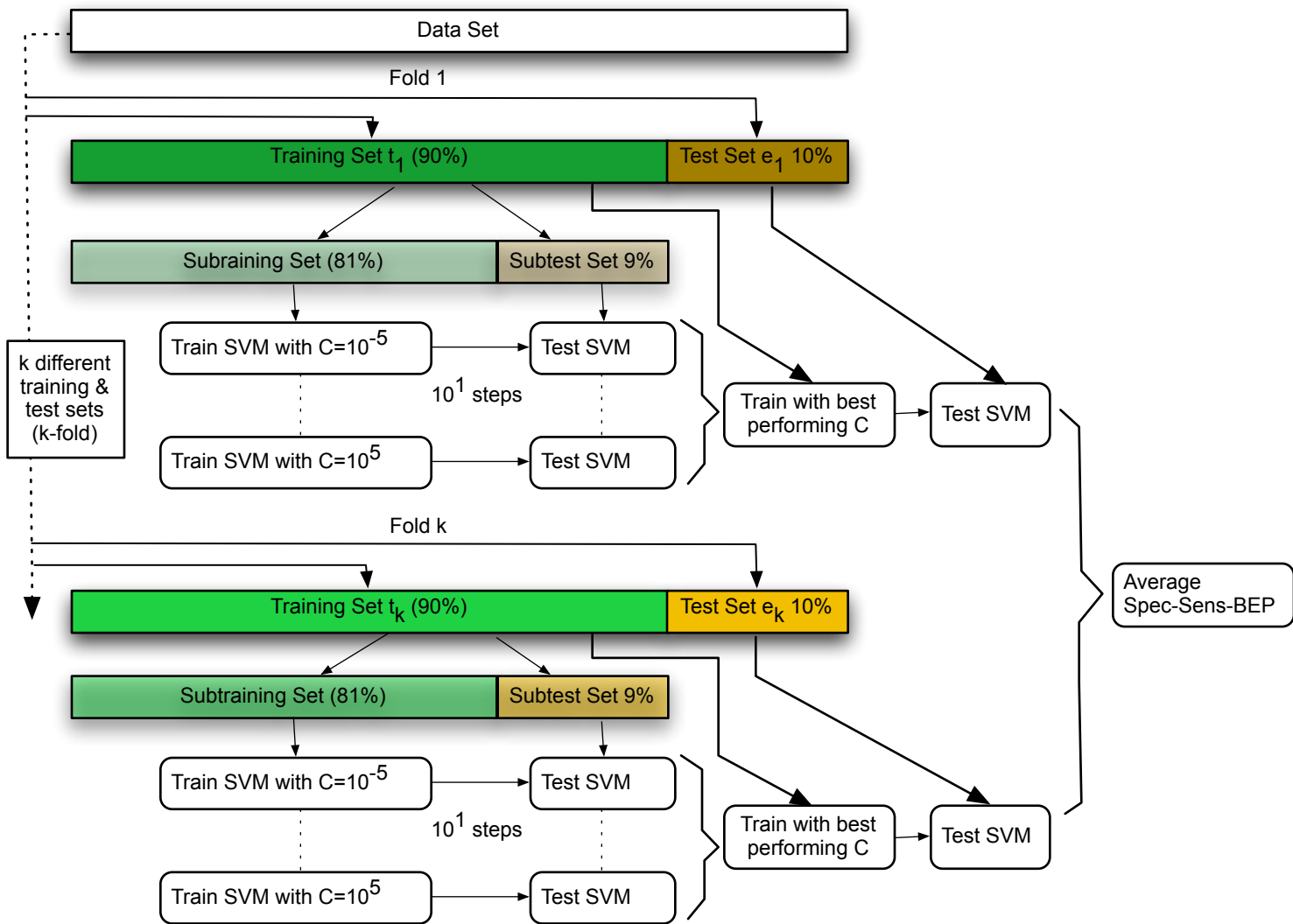

Supplement: Additional file 2 — Illustration of the k-fold cross-validation process. The positively and negatively labeled examples are split into k distinct training and test sets ti and ei, where 1 ≤ i ≤ k. To determine the best performing C value each training set ti is split into sub-training and sub-testing sets ts and es, where 1 ≤ s ≤ k. On basis of these subsets the SVM is trained several times using C values ranging from 10−5 to 105. The C value with the highest Spec-Sens-BEP is used to train the SVM with the entire training set ti. The test set ei is used to test the performance of the trained SVM by computing the Spec-Sens-BEP. These steps are repeated k times. Finally the average Spec-Sens-BEP is computed. [file 1471-2164-14-132-S2.pdf]

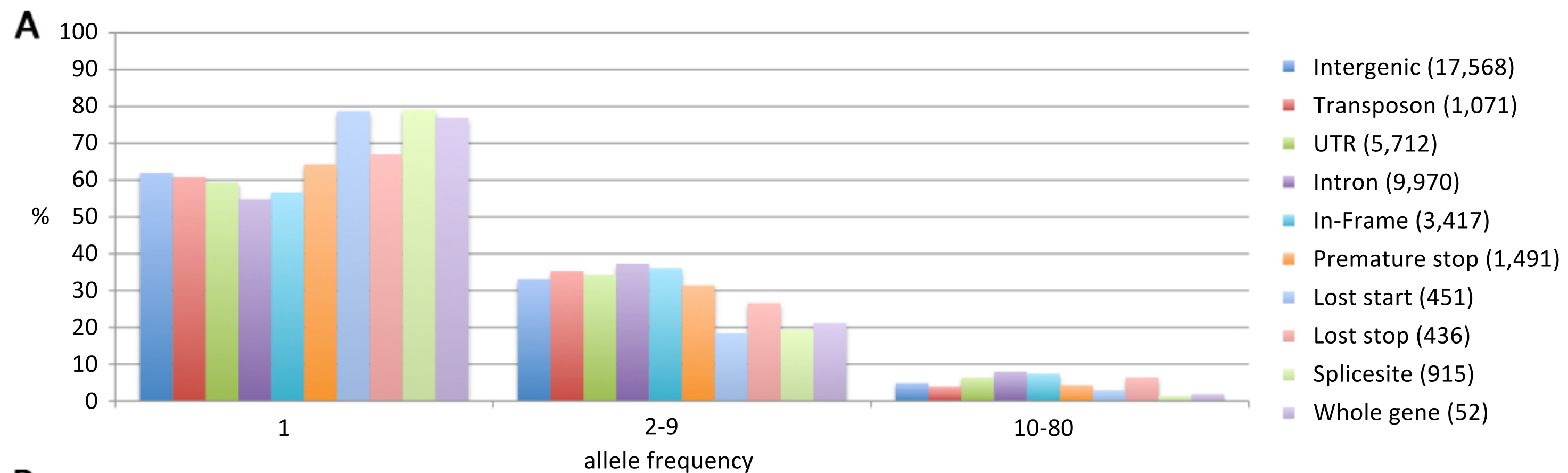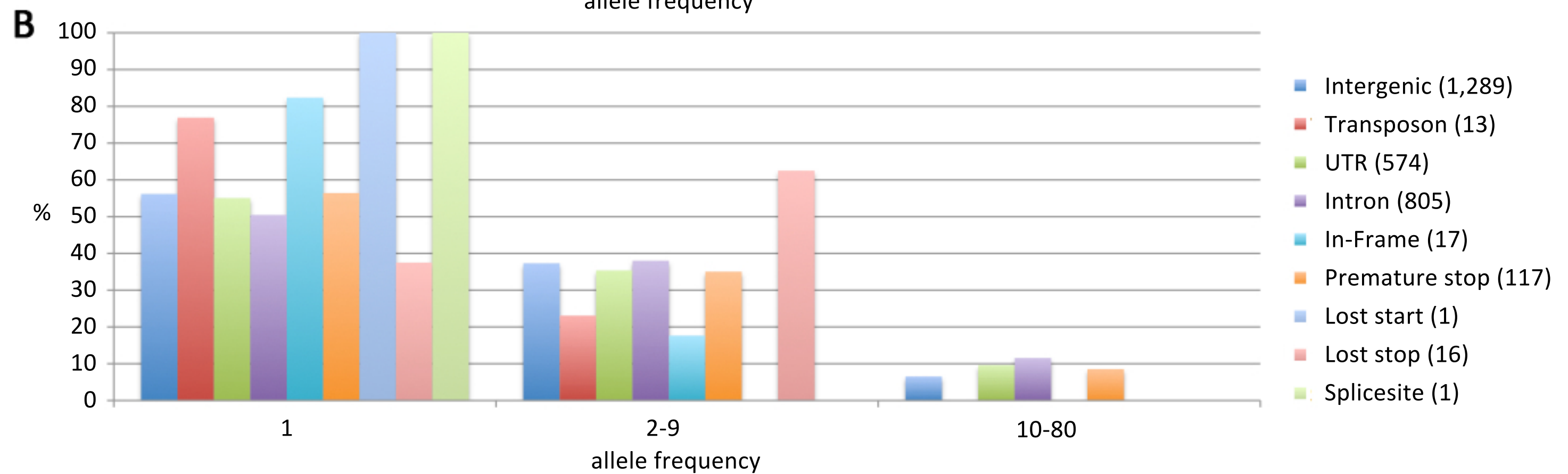

Supplement: Additional file 3 — Allele frequency of deletions and insertions in 80 genomes. The allele frequencies for deletions (A) and insertions (B), for which there was sufficient read information (see Cao et al.[2] for criteria) in all 80 strains at or 10bp surrounding the indel. They are split by functional annotation classes (obtained from TAIR8). The bars indicate the fractions of indels of each annotation class per allele frequency from all indels of the corresponding annotation class (the total number of indels in an annotation class is denoted in parentheses in the legend labels). Indels overlapping with features of different annotation classes were classified based on following priorities: CDS > UTR > intron > transposon > intergenic. Indels overlapping with coding features were classified based on following priorities: gene loss (for deletions only) > start codon change or loss > splice site change or loss > premature stop codon > stop codon change or loss > in-frame. In-frame indels do not change the frame of the coding sequence. Annotations were performed on each indel without taking into account putative compensating indels or SNPs nearby. [file 1471-2164-14-132-S3.pdf]

**A**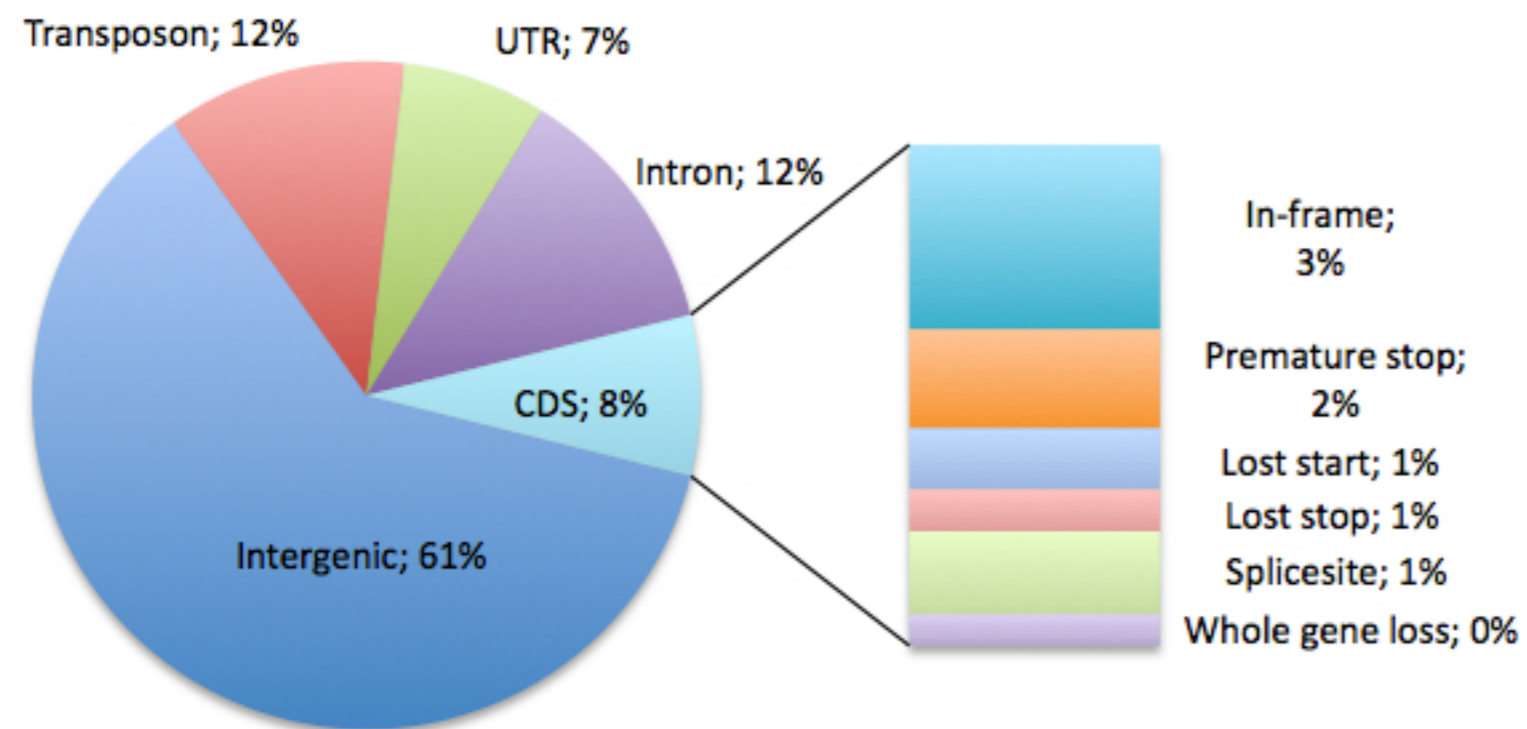**B**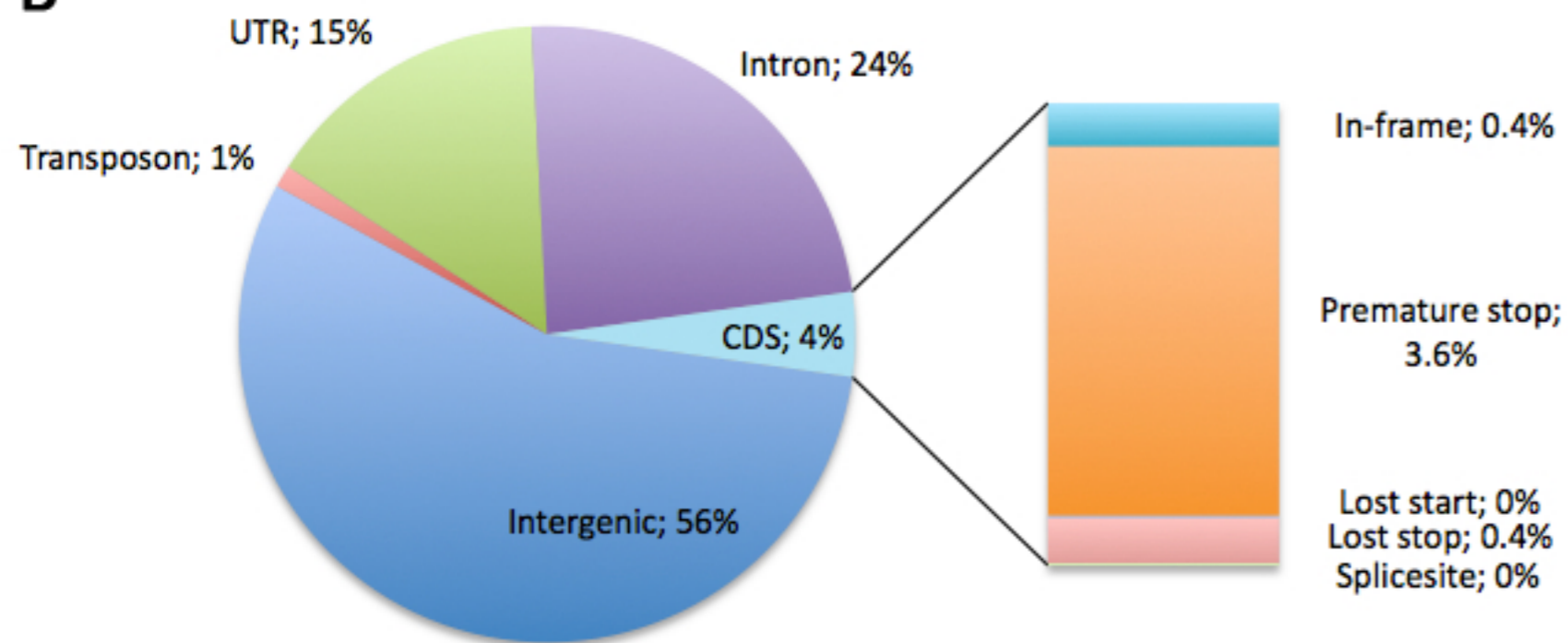**C**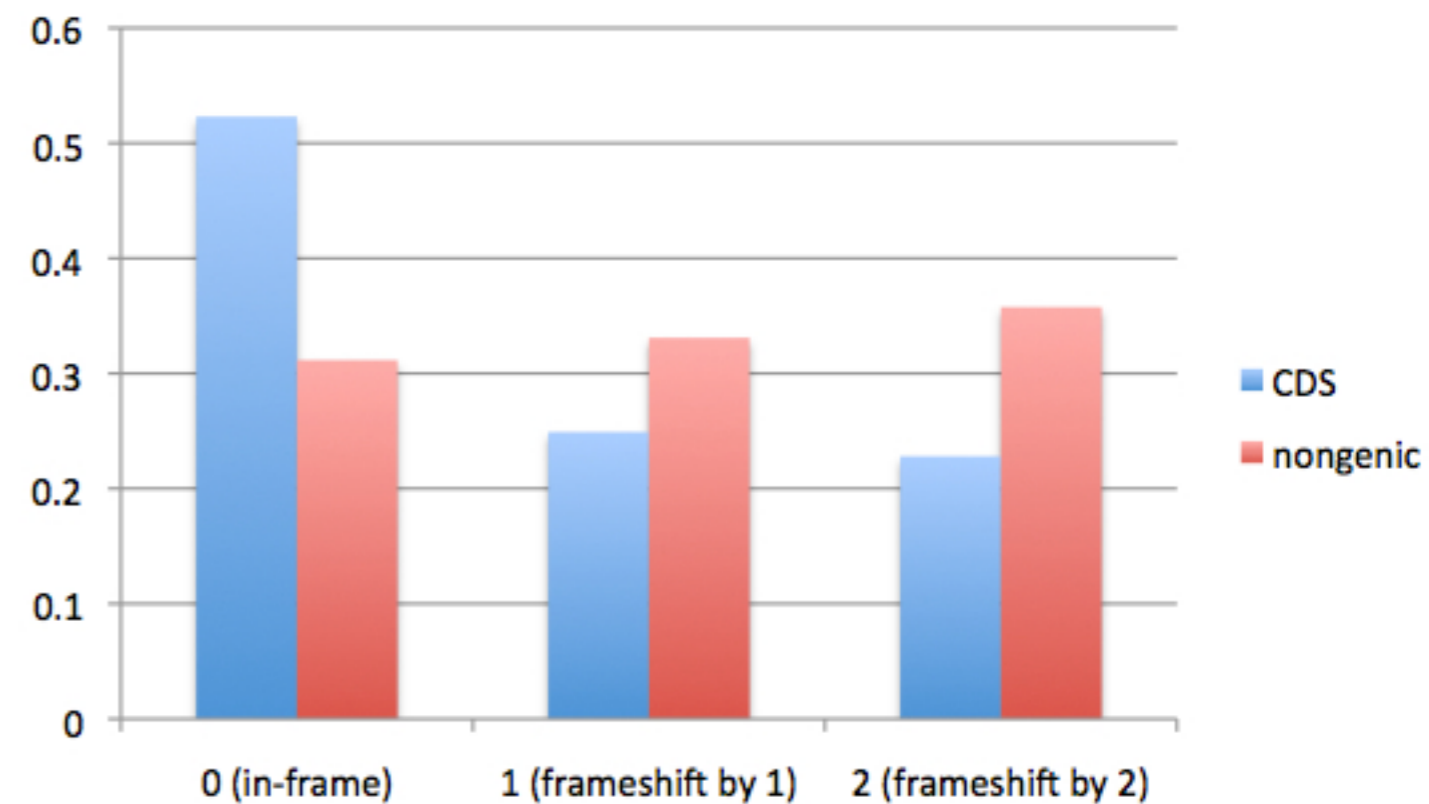

Supplement: Additional file 4 — TAIR8 annotation classes. Annotation classes of 169,246 deletions (A) and 5,500 insertions (B) in 80 genomes of Arabidopsis thaliana. For explanation of the classification scheme, see legend of Additional file 3. (C) Fractions of indels overlapping with coding sequences and overlapping with nongenic regions from all indels in corresponding classes, split by the remainder of the division of their lengths by 3. In genic regions, it is the frame of the CDS downstream of the indel. Structural variations with a length dividable by 3 in coding regions do not alter the open reading frame and are more likely to be synonymous. [file 1471-2164-14-132-S4.pdf]
